# Reviewing the Benefits of Aging Populations: Care Activities Provided by the Older People as a Commons

**DOI:** 10.3389/fpubh.2022.792287

**Published:** 2022-04-07

**Authors:** Iñigo Calvo-Sotomayor, Ekhi Atutxa

**Affiliations:** ^1^Department of Management, University of Deusto, Bilbao, Spain; ^2^Department of Finance and Economics, University of Deusto, Bilbao, Spain

**Keywords:** aging, commons, productive work, reproductive work, common good

## Abstract

This research reviews the commons paradigm as a framework for understanding the positive contribution of older cohorts to society. The commons, thought as instituting praxis that provide social goods based on autonomy and reciprocity, can help in understanding the role of this age group in providing care to the rest of society. This approach overturns the way economic literature approaches the aging phenomenon. What is understood as a “common evil”—or a problem that needs to be solved—can be analyzed as a “common good.” From this perspective, older people (population aged 65 and over) are essential to European society and the economy to carry out informal care activities that enable and facilitate the “productive” activities of working people.

## Introduction

The aging of the population is frequently regarded with great and serious alarm. However, in this paper we argue that such alarm is overstated, and that population aging can contribute positively to social sustainability.

According to Bengtson et al. (1) the most straightforward way to explain what a theory is to define it as “an attempt to explain.” Hagestad and Dannefer (2) also support this idea within the field of demographic aging. These authors promote the importance of having a theoretical framework of analysis that implies explanation. An explanation whose quality and usefulness is linked to the explanatory capacity it demonstrates over different structures and periods.

All this leads to the reflection that research involves a movement between theory and observation, between inductive and deductive cycles. In addition, there is an important connection and feedback between theories, methods, and empirical data. A process that generates “creative dissent” (2) in the world of research and keeps science moving forward.

In the field of theoretical frameworks of aging, Birren and Bengston (3) warned about the “barefoot empiricism” from which demographic analysis suffered during the second half of the 20th century. According to these authors, during this period research on aging presented analyses rich in data but poor in theoretical frameworks. The emergence, evolution, and adoption of theoretical frameworks of aging is due in part to the effort made by the aforementioned authors to promote them.

Given the need to further advance theoretical frameworks for understanding aging, this article explores the commons paradigm as a framework for understanding some of the positive contributions of population aging to society.

In order to overcome economistic and productivist views, the aim of this effort is to explore a new framework for describing, explaining and theoretically understanding the value that older people bring to our social and economic systems. A value of a deeply human nature that has profound implications for care through reproductive work that involves all the tasks associated with supporting and servicing the current and future workforce (4–6) (e.g., child bearing, rearing, and care for family members or older people as testimonies of cultural heritage and experience that are transferred from one generation to the next). One of the pillars that sustains our societies and which is often made invisible by the canonical economic indicators for measuring well-being and development (7–9).

Thus, from a multidisciplinary approach and the lens of the commons, this article aims to be the prelude—and a necessary step forward—of future applied research capable of advancing in a revisionist analysis of aging that makes it possible to point out positive aspects linked to the phenomenon.

The impetus of theoretical frameworks of aging was addressed by seminal reference works such as Emergent theories of aging (3), and subsequently expanded in others as Handbook of Theories of Aging (10–12). In addition, there have been other recent attempts to analyze the phenomenon of population aging through humanistic theoretical frameworks (13, 14).

The relevance of addressing this topic of study comes from the fact that population aging has been classified as one of the major demographic trends of the present century. A phenomenon that is accelerating (15), and that will spread to practically all countries in the coming decades (16, 17). A recent report by the US Department of Intelligence also supports these forecasts, identifying population aging as one of the major structural forces that will shape the contours of our world over the next two decades (18).

The description, analysis and understanding of the effects of aging is therefore of vital importance, and requires novel and developed theoretical frameworks. Moreover, as far as the authors of this article are aware, this is the first time that the commons paradigm is proposed as a theoretical framework to analyze part of the contribution and value provided by population aging. Specifically, the commons framework can help answer the following question: What role do European population aged 65 and over play in care as an essential input to sustain our societies?

The analysis focuses on Europe, as the world region with the highest proportion of people over 64 year old (Table 1). Even if within the group of people over 64 there are different sub-groups—younger elders, older persons, and very old people—it is important to make it clear that this is a large and highly differentiated group (with the exception of gender, since older people 64 are mostly women). Anyway, the effort to applying the commons paradigm is necessary in order to have frameworks that analyze aging from a revisionist point of view (19), bringing out positive consequences of the phenomenon, and without falling into alarmism and false beliefs. On the other hand, it is also true that there is scientific literature that presents and analyzes the aging phenomenon from a positive point of view (20–23).

**Table 1:** Evolution of the weight of people over 64 years old over the total population by world regions (%, 1950–2015).

	**1950**	**1965**	**1980**	**1990**	**2000**	**2010**	**2015**
World	5.1%	5.1%	5.9%	6.2%	6.9%	7.6%	8.3%
Africa	3.2%	3.1%	3.2%	3.2%	3.4%	3.4%	3.5%
Asia	4.0%	3.6%	4.4%	4.9%	5.8%	6.8%	7.6%
Europe	8.0%	9.5%	12.4%	12.7%	14.7%	16.3%	17.6%
Latin America and the Caribbean	3.6%	3.8%	4.4%	4.8%	5.6%	6.8%	7.6%
North America	8.2%	9.3%	11.4%	12.5%	12.3%	13.1%	14.8%
Oceania	7.4%	7.3%	8.1%	9.1%	9.9%	10.8%	11.9%

*Source: Own elaboration based on UN (17)*.

Anyway, alarmism seems to be present in certain analyses published in recent years (24–26). In this sense, Rowe (27) states that certain ideas about demographic aging must be analyzed and empirically refuted. Börsch-Supan (28) also points out that certain convictions about aging have to be scientifically analyzed in order to identify their real socio-economic challenges. The paradigm of the commons can provide an interdisciplinary and revisionist framework to achieve this goal.

This article is structured as follows; after the introduction, the following section shows the theoretical frameworks of aging, and the renewed paradigm of the commons; the third section discusses aging and reproductive work through the commons lense; finally, the article concludes with the main ideas, limitations and future avenues for research.

## Theoretical Framework

### Aging Theories: Filling the Gap

#### Current Status and Challenges of the Theoretical Frameworks of Aging

Over the last decades, there has been a resurgence of interest in the different theories that can be used to understand aging (10–12). These publications examine the development of theoretical and conceptual frameworks of the aging phenomenon. A process defined as “dynamic, multifaceted, and interactive” (29).

While analyzing the evolution of aging theories, significant challenges are identified in structuring and articulating different theoretical frameworks. Research on aging theoretical frameworks is growing in relevance and currency, but it still presents certain challenges (13), among them:

Overcoming “level myopia” (or obsession with micro aspects)

Hagestad and Dannefer (2) call “level myopia” to the excessive micro-focus on the individual that theoretical frameworks on aging sometimes adopt. A tendency that limits their explanatory power. Gans et al. (30) share this concern and defend the development of aging theoretical approaches that achieve a connection between the individual and the context or—as Bass (31) puts it—that overcome the tension between structure and agency.

The importance of the interdisciplinary approach

Bengtson and Settersten (10) and Bengtson et al. (12) call it one of the most relevant trends in theoretical frameworks on aging, as many authors studying aging advocate for cross-pollination and the use of different perspectives, theories, and knowledge to understand the phenomenon. Collaboration between different disciplines is therefore essential. The National Academy of Sciences (32) also adopts this interdisciplinary approach.

The search for a unified theory

Bengtson and Settersen (10) point out that during the last decades, not only have interesting approaches and theoretical lines proliferated in different fields, but also general and unifying theories are beginning to be sought. Gavrilov and Gavrilova (33), in their search for a general biodemographic theory of aging, or the proposal by Bass (31) to generate an integrative theory of gerontology are examples of progress in this direction.

#### Aging Theories

In addition to presenting challenges, aging is a broad and multifaceted field of study, and five broad areas classify the different theoretical approaches to this population phenomenon (10, 11):

Biological theories of aging: this branch of aging theories analyses the aging process through a biological approach. Some of its theories are evolutionary theories, those related to genetics or the study of aging in invertebrates (34).

Psychological theories of aging: Psychological theories of aging address how cognitive ability, social relationships, emotion regulation or personality are influenced by aging (35).

Social science theories of aging: This area of theories emphasizes aging as a social process (36). Relevant theories in this field are, for example, the life course perspective or those addressing the relationship between inequality and aging.

Societal and public policy theories of aging: This typology of theories aims to explain the complex interrelationship between public policy-making, social processes and the well-being of older people (1). These include those addressing demographic aging and the welfare state (37), or those linked to the political economy of aging (38).

Interdisciplinary theories of aging: This last area of theories connects different fields of knowledge. Interdisciplinary theories have started to emerge in this field, such as the biopsychosocial understanding of healthy aging or biodemography (39).

## Discussion: Aging and Reproductive Work Through the Commons

### Embracing a Revisionist Perspective

Population aging is one of the greatest human achievements, and a common good of humanity that can be considered as a demographic success story, since this phenomenon is associated with the shaping of welfare states, socioeconomic development or progress in medical technologies, among other issues (17, 40).

Nevertheless, the economic literature analyzes population aging from a negative perspective, and as a problem to be solved (14). As mentioned above, as a “common evil.” In this line, there are numerous negative references to aging. Concepts such as “demographic burden” (19, 41), “demographic time bomb' (42, 43), or “age war” (41) are not rare in the scientific literature.

Undoubtedly, changes in the demographic pyramid have social and economic effects (44), so it is evident that population aging causes diverse and broad socioeconomic challenges (45–47). However, we fall back into a “level myopia” if we are not able to understand aging and the social, cultural, and economic capital accumulated by the older cohorts as a fundamental pillar for sustaining our societies.

If aging is understood as a common good, it seems consistent not to adopt an “alarmist” perspective from the point of view of changes in the demographic structure. Actually, alarmist predictions regarding, for example, the population explosion during the 20th century never materialized (44). Therefore, Kelley (19) argues that during the last decades there has been an evolution in demographic research from an “alarmist” to a “revisionist” position, which has led to a less apocalyptic debate and a broadening of the focus of description, analysis, and understanding of demographic phenomena. The paradigm of the commons deeps into this conception of aging. It is not a “common evil” of lesser consequences, but a “common good” necessary to provide the informal care—understood as the assistance given by a member of the social network (48)—that sustains the productive system.

In this line, the National Research Council of the USA (49) establishes a non-alarmist or “revisionist” position when carrying out analyses in relation to population growth. This “revisionist” perspective relies on three assumptions that can be applied to analyze the aging phenomenon from the commons paradigm:

Focus on the medium and long term: Social capital is predominantly significant in the elderly population (50). Older people transfer cultural and social wealth to new generations, which is necessary to project the future of society.

Evaluation of multiple positive and negative effects: It is necessary to go beyond the economic and productivist effects of aging. In addition to the economic wealth produced, societies are institutions that are home to other typologies of wealth (such as social and cultural). Older people play a central role in generating and maintaining different kind of wealth.

Consideration of the indirect effects of demographic changes: The progressive women's entry into the labor market, together with the greater presence of older people in the population pyramids, seems to be showing a certain transfer of informal care to this latter group. These social and economic processes need to be monitored in order to understand the contribution of older people to the prosperity of our societies.

These assumptions make the research agenda of this school very demanding, as it takes into account a wide variety of forces and a broad time period to carry out its analyses and contrasts. This means that the conclusions of the “revisionist” school are more moderate than those of the “alarmist” school. An important input for demographic analysis in recent decades.

In this sense, it is important to highlight that there are authors who defend a non-alarmist view and an understanding of population aging from a more sensible perspective (14, 28, 51–53).

### Positive Aspects of Aging Through the Commons

Rowe (53) not only considers it of interest to adopt a non-alarmist approach to research on population aging, but also to analyze possible positive consequences and potential of the great demographic transformation that aging entails. Specifically, this author advocates recognizing “the important positive contributions and potential productivity of an aging society” (53).

It is precisely at this point that the paradigm of the commons makes its contribution. *Community-based resource management* (54), *common goods governance* (55), *common democracy* (56), *procommons* (57), and *common instituting praxis* (58) are conceptualisations that, recurrent in time, have strived to demonstrate models of community-based governance of social goods that differentiate from those from the public-state and private-market as we understand them. Overlooking the paternalistic relationship with the public and/or the blind acceptance of market forces, the commons reveal the possibilities and potential of collective self-organization for sustainable management of different social goods, such as de provision of reproductive work. The paradigm of the commons includes all (self)-transformative activities, performed by subjects that are able to create new institutions in search of higher degrees of human autonomy (59, 60). In fact, “the so-called common goods are not just goods; they are not “things” that are separate from us, they aren't even just shared goods. (…). They are social practices of commoning, based on the principles of sharing, caring and producing jointly” (54). In other words, it is a decision to self-organize in order to manage and provide a good based on solidarity and reciprocity [(61, 62), p. 10].

Thus, the older people who provide the necessary reproductive work can be considered as Commoners, that is to say, members of a community who, through the care of the rest of the people in their environment, make it possible to sustain productive work and, consequently, the system itself. Specifically, it should be recognized that positive contributions to society are not only made through the improvement of the factors of production productivity. Moreover, the behavior of this productivity depends on the response of other dimensions that are vital for the shaping of prosperous societies. This is the case of informal care which, as described above, is increasingly provided by the elderly.

The previous statement—i.e., thinking about the contribution of the elderly to caring activities from the paradigm of the commons—, deeply challenges the widespread myth in the literature and public debate that older people are less productive (27, 52). A vision anchored in the past that does not take into account the phenomenon of increased longevity and “compression of morbidity,” which points out that globally people are extending their lives more fully and productively (63). A process that generates cultural and social wealth and the sustenance of informal care, as discussed above.

Today, there is an open debate about whether aging influences economic productivity (64). A debate that, moreover, is divided between research that defends a significant and negative relationship (65–67) and, on the other hand, authors who do not believe that such a relationship exists (28, 52, 68–70). However, the authors of this research believe that the debate on the wealth generated by older people must go beyond the purely economic. In order to understand the role that older cohorts play in our societies, it is necessary to broaden our understanding of prosperity and development.

Indeed, if we go down this path we will be able to recognize that older people have shown to be more providers than receivers of support up to the age of 80 (52). In other words, older people continue to generate wealth in social and family terms well-beyond the age of 65. Along these lines, Durán (4) notes that older people have “a role in the production and consumption of non-monetary services within households” as well as an important role in the production of volunteer services. These contributions can be exemplified by the fundamental contribution—both monetary and care—of older people to their children and grandchildren after the severe economic crisis in Spain in 2008, or the research about how older people are potentially very important contributors in local voluntary organizations (71). As shown in Table 2, Spain has a significant population over 64 years of age, and a progressive aging of the different groups within this age cohort (younger elders, older persons, and very old people).

**Table 2:** Evolution of the weight of people over 64 years old over the total population of Spain (%, 1950–2015).

	**65+**	**70+**	**75+**	**80+**	**85+**	**90+**
1950	7%	4%	2%	1%	0%	0%
1960	8%	5%	3%	1%	0%	0%
1970	10%	6%	3%	2%	0%	0%
1980	11%	7%	4%	2%	0%	0%
1990	13%	9%	6%	3%	1%	0%
2000	17%	12%	7%	4%	2%	1%
2010	17%	13%	9%	5%	2%	1%
2015	19%	14%	10%	6%	3%	1%

*Source: Spanish Statistical Office – INE*.

From this perspective, older people are essential to society and the economy, as they carry out “reproductive” activities that enable and facilitate the “productive” activities of working people. These activities are essential to generate public trust, which in turn is the basic fuel that runs the economic machinery (72). This idea is also supported by Durán (4) who states that “if employed relatives of dependents can remain in their occupation, it is precisely because the burden of care is mostly assumed by other relatives outside, and in many cases expelled from, the labor market, among whom housewives and retired people play a key role.”

## Conclusions

Theoretical frameworks on aging are constantly evolving and present important challenges to further unravel the multifaceted phenomenon of population aging. In this context, the search for new frameworks that overcome level myopia and provide a multidisciplinary perspective is particularly relevant.

The paradigm of the commons responds to these challenges, and establish itself as a new theoretical framework that provides a holistic and multilevel view of demographic aging and its positive contributions. Within the classification of theoretical frameworks enunciated in this research article, it can be classified under the heading of Societal and public policy theories of aging. Besides, the lens of the commons can help to understand this demographic phenomenon through a non-alarmist lens, and shed new light on the understanding of aging. In particular, it can be of great help in understanding that, rather than a “common evil.” Aging is not only one of the greatest human achievements, and a demographic success story, but also ha phenomena that continues providing essential a “common goods” for the sustainability of our societies and economies.

In addition to conventional narratives that are mainly oriented to economic wealth, the paradigm of the commons allows us to understand that there are social goods that must be protected in order to sustain our societies. There are other social and cultural wealth that condition the prosperity of territories, and that are contained in the older cohorts. This is the case of informal childcare, which is increasingly provided by this segment of the population, mostly since the incorporation of women into the labor market.

This article, as a “perspective” manuscript, offers a prelude to the advancement of scientific knowledge on the social, cultural, and economic value created by the elderly. In fact, it is a contribution in itself the multiple doors this paper opens to future research on, among others, the gender perspective also in the care provided by the elderly, or the involvement in these activities conditioned by the cultural and social divergences of the different regions of the globe. All in all, in the face of alarmist perspectives focused mainly on the potential negative economic impacts of aging, it is crucial to advance in the development of empirical research capable of analyzing the social wealth provided by older people in a more comprehensive and sophisticated way. The paradigm of the commons offers a useful conceptual framework for identifying other social and cultural values contained in—and provided to—society by the older cohorts. Only by understanding the relevance of these values for the preservation of our economic systems, we will be able to move toward an integral human and sustainable development.

## Data Availability Statement

The original contributions presented in the study are included in the article/supplementary material, further inquiries can be directed to the corresponding author/s.

## Author Contributions

Both authors listed have made a substantial, direct, and intellectual contribution to the work and approved it for publication.

## Funding

This paper has been financially supported by Deiker of the University of Deusto.

## Conflict of Interest

The authors declare that the research was conducted in the absence of any commercial or financial relationships that could be construed as a potential conflict of interest.

## Publisher's Note

All claims expressed in this article are solely those of the authors and do not necessarily represent those of their affiliated organizations, or those of the publisher, the editors and the reviewers. Any product that may be evaluated in this article, or claim that may be made by its manufacturer, is not guaranteed or endorsed by the publisher.
